# Huge Left Atrial Myxoma Presenting as Pulmonary Embolism on a CT Pulmonary Angiogram

**DOI:** 10.7759/cureus.98031

**Published:** 2025-11-28

**Authors:** Abdul Nadim Asil, Youssef Abouelela, Alberto Albanese

**Affiliations:** 1 Cardiothoracic Surgery, Basildon University Hospital, Basildon, GBR

**Keywords:** cardiac tumour, ct pulmonary angiogram (ctpa), large atrial myxoma, pulmonary angiogram, pulmonary embolism (pe), transesophageal echo

## Abstract

Atrial myxomas are the most common benign primary cardiac tumours. Despite their benign nature, they can significantly increase morbidity and mortality. The majority occur in the left atrium, although familial myxoma syndromes may present in atypical locations. Patients may present with symptoms related to intracardiac obstruction or embolic phenomena or remain asymptomatic. A 76-year-old woman presented to her general practitioner with shortness of breath and features consistent with pulmonary embolism. Computed tomography pulmonary angiography (CTPA) revealed a large filling defect within the left atrium. Subsequent transthoracic echocardiography confirmed the presence of a large left atrial myxoma attached to the atrial base. Intraoperative transoesophageal echocardiography (TOE) further demonstrated the attachment site. The patient underwent successful surgical excision via a median sternotomy and trans-septal approach. Several modalities are available for diagnosing left atrial myxomas, with TOE providing excellent diagnostic accuracy. However, CTPA can also detect atrial myxomas with the additional advantage of being non-invasive. While TOE remains the preferred modality for diagnosis of left atrial myxomas, CTPA can be a useful complementary investigation. It may help distinguish atrial myxomas from pulmonary embolism, as demonstrated in this case, while offering the added benefit of being non-invasive. It is extremely useful for differentiating atrial myxomas from pulmonary embolism, as discussed in the case, with the added benefit of being non-invasive.

## Introduction

Atrial myxomas are the most common tumour of the heart, representing 50% of all benign cardiac tumours in adults and 15% of cardiac tumours in children. Specifically, 75% of myxomas are found in the left atrium and less frequently in the right atrium, right ventricle, and left ventricle [1].

Pulmonary embolism and atrial myxomas may produce symptoms of shortness of breath and hypotension, albeit in slightly different mechanisms. In the former, there is reduced cardiac output from right heart strain, as there is a blocked pulmonary blood flow. In contrast, atrial myxomas are the cause of reduced cardiac output as there is a physical obstruction.

This case report was previously presented as a poster presentation at the 2023 SCTS (Society for Cardiothoracic Surgery) Annual Meeting on March 20, 2023.

## Case presentation

We present the case of a 76-year-old Caucasian woman who developed shortness of breath with features suggestive of pulmonary embolism. She was initially assessed by her general practitioner (GP). The GP found the patient to be slightly tachycardic with a heart rate of 105 bpm, hypotensive with a systolic blood pressure (BP) of 100 mmHg. The GP advised the patient to attend the hospital to complete a computed tomography pulmonary angiography (CTPA) to exclude pulmonary embolism. The CTPA revealed an incidental finding of a large left atrial myxoma measuring approximately 7 cm × 7 cm × 6 cm.

Her past medical history included lung parenchymal disease with preserved pulmonary function, despite being an ex-smoker. She also had a small patent foramen ovale (PFO), mild mitral regurgitation with a transvalvular gradient of 2 mmHg, preserved biventricular function, and no evidence of coronary artery stenosis. Additionally, she had a background of urinary incontinence. The patient reported no family history of cardiac myxoma.

**Figure 1:**
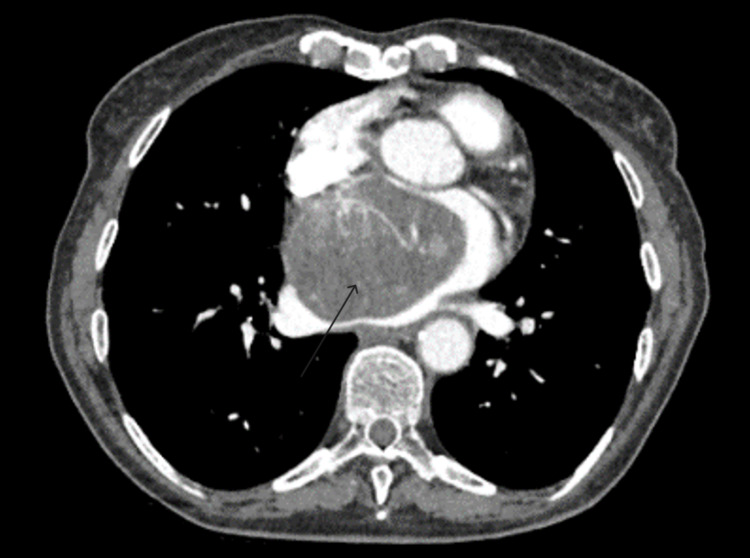
CTPA showing filling defect in the left atrium Arrow pointing to the atrial myxoma; CTPA: computed tomography pulmonary angiography

The patient was referred to our centre for surgical excision of the atrial myxoma. CTPA (Figure 1) demonstrated a large filling defect in the left atrium with a well-defined outline, consistent with a left atrial myxoma. Transthoracic echocardiography further confirmed the diagnosis.

**Figure 2:**
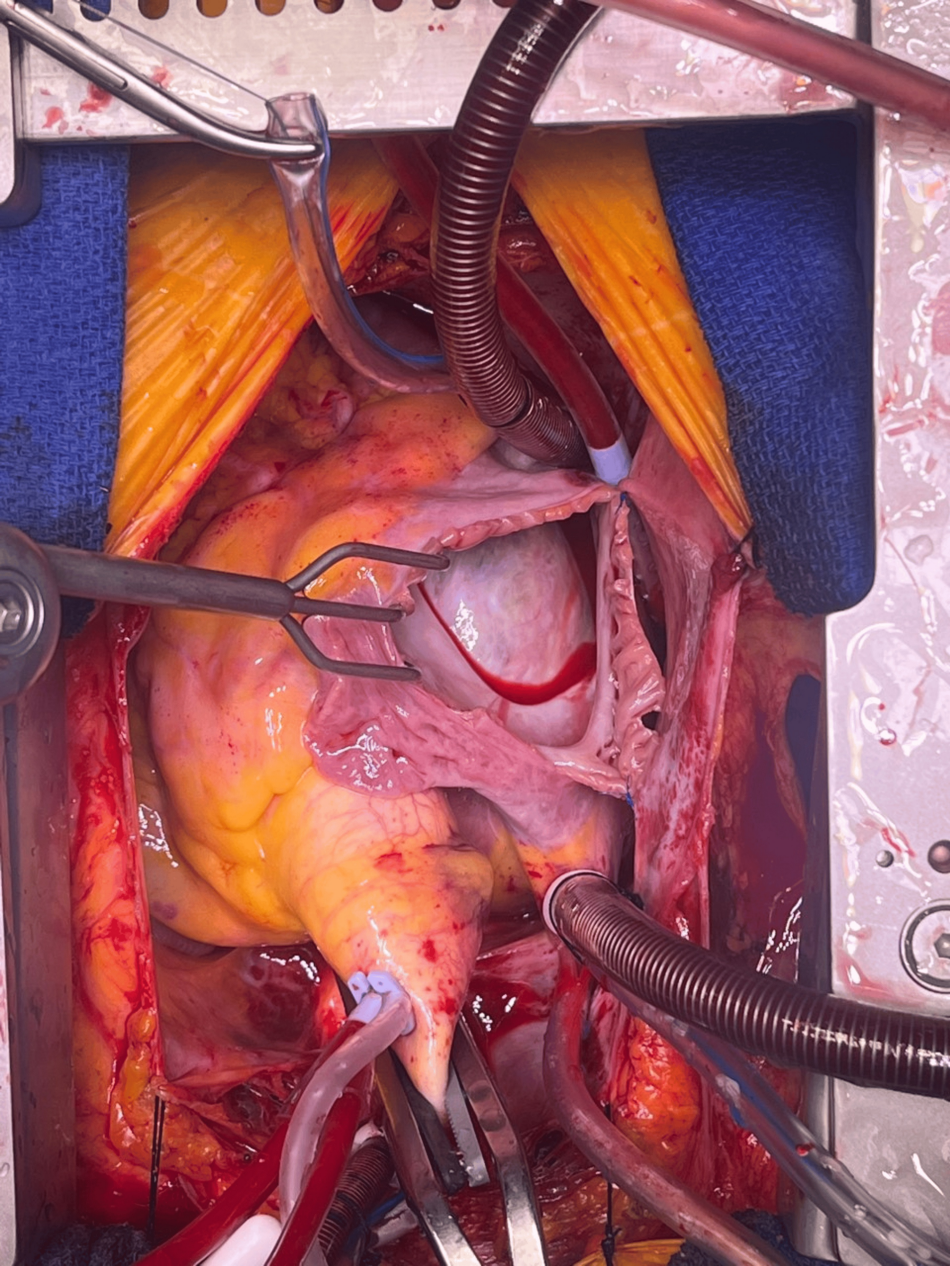
Trans-septal approach with central cannulation

She was evaluated by the cardiothoracic team and scheduled for surgical excision via a trans-septal approach through the right atrium (Figure 2). Preoperative transoesophageal echocardiography (TOE) delineated the margins of the tumour. A routine median sternotomy was performed, and cardiopulmonary bypass was established with central cannulation. Cardioplegia was delivered through the aortic root. Upon opening the right atrium, the interatrial septum was noted to be bulging into the right atrial cavity due to the size of the tumour. The myxoma was excised by enucleation of the mass along with resection of its septal attachment after opening the left atrium. The attachment zone was excised and closed directly. The resultant septal defect was repaired with a pericardial patch using 5-0 Prolene. The right atrium was then closed in standard fashion.

The patient remained haemodynamically stable throughout the procedure. In the immediate postoperative environment, she developed atrial fibrillation, which was chemically cardioverted. She recovered well and was discharged home on day five postoperatively in good general health.

**Figure 3:**
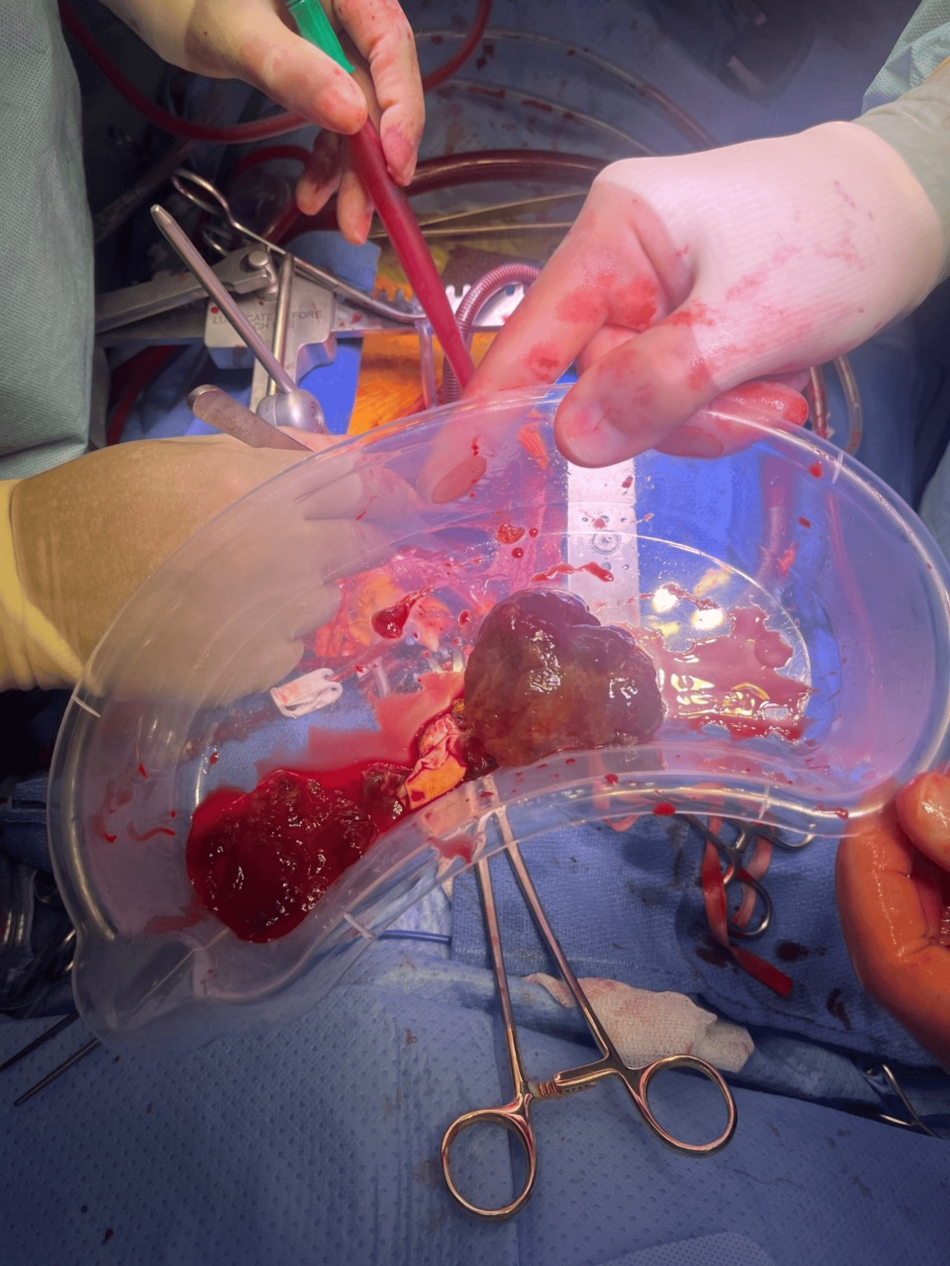
Excised myxoma

**Figure 4:**
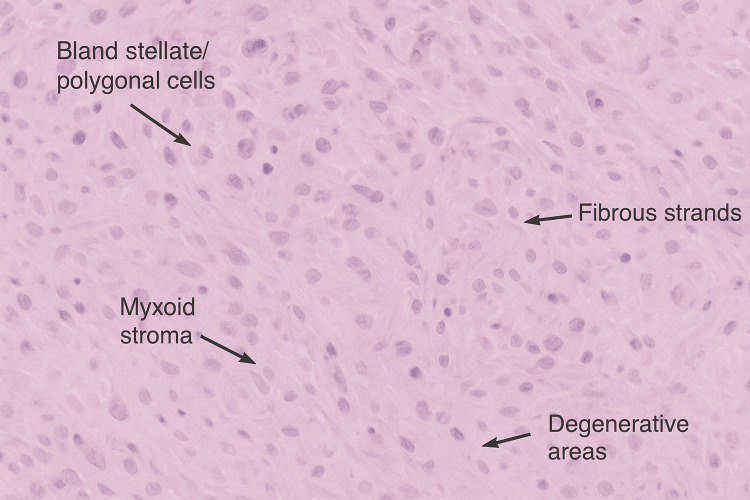
Excised myxoma histopathology Myxoid stroma with degenerative areas and bland stellate cells

Gross examination of the excised specimen revealed a homogenous, gelatinous mass with hemorrhagic areas on cut section. The fragments weighed 132 g (Figure 3). Histological analysis showed bland polygonal cells within a mixed myxoid and fibrous stroma, often forming trabeculae. Degenerative changes, including calcification, thrombus, fibrin, and haemosiderin deposition, were noted, confirming features of myxoma (Figure 4). There was no evidence of granuloma formation or malignancy. Further tumours were excluded by TOE. The findings were consistent with a primary sporadic left atrial myxoma.

## Discussion

Diagnosis

Initially, a thorough history from the patient can help point towards a diagnosis of atrial myxoma. The most common symptom of left-sided myxoma is dyspnoea, and the least common symptom is chest pain. The mean duration of symptoms is approximately 10 months [2]. This case is of a large atrial myxoma, which, in general, is more likely to present with constitutional symptoms and congestive heart failure [2]. Progressive exertional dyspnoea (New York Heart Association (NYHA) grading class III) was the only symptom in this lady that may allow clinicians to consider myxoma as a differential, although this is less likely to be a large atrial myxoma, as the patient lacked the symptoms of congestive heart failure or constitutional symptoms. Right-sided myxomas tend to present similarly but also resemble symptoms of right-sided heart failure or tricuspid regurgitation [3].

The role of transoesophageal echo in diagnosing and evaluating atrial myxoma is well documented. It offers a comprehensive understanding of the anatomical environment and structure of the mass as well as any functional abnormalities [4]. In this patient, it was used preoperatively to confirm the diagnosis and intraoperatively to monitor cardiac function. It plays a key role in diagnosing and assessing intracardiac masses as per the UK guidelines.

The CTPA can also help indicate if there are flow obstructions in the heart and surrounding vessels. The main advantage is that it is non-invasive and quick. The atrial myxoma was detected as an incidental finding on CTPA. This imaging modality can detect a range of pathologies, but definitive diagnoses may require further investigation and evaluation [5-7]. In the case discussed, the CTPA is useful as it can differentiate between pulmonary embolus and left atrial myxoma.

Intervention and outcome

Atrial myxomas are associated with stroke (from embolisation of the mass); therefore, it is imperative to consider surgical removal. The irregular surface and increased size of the tumour carry an increased risk of embolisation [8]. Non-myxomatous emboli may appear similar to myxomas in echocardiography, but the key difference is that myxomas tend to have a stalk structure on imaging, which can be used to distinguish between the two similar structures. However, myxomas pose a similar threat of embolisation and stroke, like that of emboli in the heart. Invariably, the symptoms and clinical presentations of the stroke will depend on the location of the emboli. In addition, atrial myxomas with concomitant atrial fibrillation can increase the risk of stroke [9].

Outflow tract obstruction is uncommon with atrial myxoma and may be more likely with ventricular myxomas. Additionally, mitral and tricuspid stenosis or regurgitation are recognised complications of myxomas, in which case, the patient may develop symptoms such as dyspnoea or syncope. As Buksa et al. found, there can be ‘early diastolic tumour plop’ sounds, which can mimic valvular abnormalities [10].

Myocardial infarction (MI) is an uncommon complication of atrial myxoma; there have been a few reported cases of infected left atrial myxomas, which have subsequently caused MI [11,12]. However, it is common for embolisation to occur (incidence of 30-40%), albeit embolisation to the coronary vessels causing MI/ischaemia is rare too [13].

Prompt surgical resection is the treatment of choice to minimise the risks outlined above. In some cases, early surgical intervention has proven to be more beneficial [14]. Biatrial access allows the surgeon to completely excise the mass and enables a better view of the chambers of the heart. The intervention is for curative intent and can result in excellent outcomes in post-surgical survival outcomes [15,16].

Post-surgical complications can include wound infection, bleeding, cardiac arrhythmias - including atrial fibrillation. Late-onset atrial fibrillation is common in patients postoperatively [16]. The predictor of mortality is linked more to the histology of the mass and duration of time the patient is on cardiopulmonary bypass [17] as opposed to any of the surgical complications mentioned above. Furthermore, the recurrence of myxomas is more likely in the first decade postoperatively and requires further follow-up and imaging surveillance, especially if the patient is young [18].

## Conclusions

Although transoesophageal echocardiography remains the most accurate diagnostic modality for left atrial myxomas, other radiological techniques such as CTPA can be extremely valuable. In particular, CTPA can aid in differentiating atrial myxomas from pulmonary embolism, as demonstrated in this case, while offering the additional advantage of being a non-invasive investigation.
